# Severe colchicine intoxication after self-administration of colchicine concomitantly with loxoprofen

**DOI:** 10.1007/s00540-012-1531-2

**Published:** 2012-12-08

**Authors:** Akinori Yamazaki, Hiroshi Iranami, Koichi Nishikawa

**Affiliations:** 1 Department of Anesthesiology, Wakayama Medical University, 811-1 Kimiidera, Wakayama City, Wakayama 641-0012 Japan; 2 Department of Anesthesiology, Japanese Red Cross Society Wakayama Medical Center, 4-20 Komatsubara-dori, Wakayama, Wakayama 640-8269 Japan

**Keywords:** Colchicine, Intoxication, Loxoprofen, Multiple organ failure

To the Editor:

We describe a patient with gout who developed serious multiple system organ failure after the self-administration of colchicine together with loxoprofen.

A 38-year-old male (height 175 cm, weight 75 kg) with no pre-existing diseases had been prescribed colchicine (1 tablet) together with loxoprofen (1 tablet) for gout attack by his family physician. Following the severest pain attack at his toe that he had ever experienced, he self-administered 30 tablets of colchicine (15 mg) together with ten loxoprofen tablets (600 mg) within a 6-h period. His pain subsided 3 h after the last intake of the tablets, but he presented to our medical center because of gastrointestinal symptoms including nausea, vomiting, diarrhea, and hematemesis.

The patient developed acute renal failure on the second hospital day, and on the third hospital day, he entered a delirious state, with thrombocytopenia, respiratory failure, and severe hypotension. Granulocyte colony stimulating factor (250 μg/day) was administered for his severe pancytopenia on hospital days 4–7. On hospital day 9, his urine output began to increase, and on hospital day 18, the patient had been successfully weaned from the mechanical ventilation. He was discharged from the hospital on hospital day 26. All of the blood and laboratory parameters on hospital day 25 were within the normal ranges, but alopecia, muscle weakness (MMT 4/5), disappeared ankle tendon reflex, reduced evoked potentials of peripheral nerves, including the median, ulnar, and tibial nerves, remained (Table 1).

**Table 1:** Blood, serum, and coagulation data during the hospitalization of this patient

Blood, serum, and coagulation data	Normal range	Day 1	Day 2	Day 3	Day 4	Day 5	Day 6	Day 7	Day 18	Day 25
White blood cells (/ml)	4000–7000	29700	19500	6000	900	400	900	3500	11400	8400
Hemoglobin (g/dl)	14.0–18.0	17.6	16.1	14.4	11.0	9.8	7.9	7.3	8.6	8.7
Hematocrit (%)	40.0–52.0	51.7	47.8	42.5	31.7	27.3	22.6	20.5	24.8	25.7
Platelets (/ml)	15.0–40.0	26.7	11.0	2.7	2.3	1.5	1.3	2.0	10.2	28.1
Prothrombin time (%)	85–120	36	45	76	87	87	93	95	77	
Prothrombin time (INR)		2.3	1.89	1.21	1.10	1.10	1.05	1.03	1.20	
Activated partial thrombin time (s)	28–40	56.4	70.3	52.0	47.9	38.7	41.7	40.6	37.6	
Fibrinogen (mg/dl)	200–400	374	598	799	766	822	692	586	396	
Alkaline phosphokinase (U/l)	104–338	2579	2714	1323	565	481	359	327	179	229
Aspartate amino transferase (U/l)	5–40	375	405	344	210	188	177	115	80	29
Alanine amino transferase (U/l)	5–35	52	49	42	44	77	100	79	44	31
Lactate dehydrogenase (U/l)	50–400	12250	13630	10740	4850	2083	1115	835	1290	506
Creatinine phosphokinase (U/l)	20–190	1058	1518	2871	1937	929	283	383	799	36
Blood urea nitrogen (mg/dl)	8–20	42	41	33	28	25	30	32	80	29
Creatinine (mg/dl)	0.6–1.5	4.4	3.8	3.5	2.7	2.8	2.6	2.2	2.8	0.9
C-reactive protein (mg/dl)	−0.3	35.51	53.88	54.25	26.85	25.51	19.05	21.05	2.72	0.95

The first symptom of this patient was that of gastrointestinal tract, followed by the subsequent multiple organ failure, including the cardiovascular, renal, hepatic, respiratory, myeloproliferative, central and peripheral nervous systems, which sequentially developed as precisely described in Stapczynski et al.’s review [1]. The severity of our patient’s colchicine intoxication correlated well to the intake doses; i.e., an oral intake of <0.5 mg/kg only exerts gastrointestinal tract symptoms, that from 0.5 to 0.8 mg/kg results in multiple organ failure, and an oral intake of >0.8 mg/kg can be lethal [2]. The total intake of colchicine in this patient was 15 mg (0.2 mg/kg), and he did not have any pre-existing diseases. The co-administration of macrolides promotes colchicine’s toxicities [3, 4], but, to our knowledge, there has been only one report of colchicine toxicity in association with indomethacin [5]. Colchicine, anti-inflammatory drugs (NSAIDs), and corticosteroids are commonly used to treat gout. It would appear that a possible interaction between colchicine and NSAIDs could cause a fatal complication.
